# Overexpression of EGFR and TGFα in von Hippel–Lindau-Related Central Nervous System Hemangioblastomas

**DOI:** 10.3389/fonc.2020.00703

**Published:** 2020-05-05

**Authors:** Zhen Liu, Liang Li, Zhiqiang Yi, Hongzhou Duan, Runchun Lu, Chunwei Li, Jingcheng Zhou, Kan Gong

**Affiliations:** ^1^Department of Neurosurgery, Peking University First Hospital, Beijing, China; ^2^Department of Urology, Peking University First Hospital, Beijing, China

**Keywords:** VHL disease, genetic mutation, hemangioblastomas, EGFR, TGFα

## Abstract

**Background:** Central nervous system (CNS) hemangioblastomas (HGBs) are the most frequent cause of mortality in patients with von Hippel–Lindau (VHL) disease. Characteristics of multiple and recurrent disease cause certain difficulties in the treatment of CNS HGBs.

**Methods:** VHL-related HGB cases treated surgically at our hospital from September 2015 to February 2019 were analyzed. Patients meeting the clinical diagnostic criteria underwent genetic testing. Real-time PCR and immunohistochemistry were used in HGBs to verify differential expression of mRNAs and proteins, respectively. Furthermore, correlations between the differentially expressed proteins and the histological grading, genetic mutations, and tumor burden were also analyzed.

**Results:** A total of 21 patients with VHL syndrome confirmed by genetic testing (missense group, 9; partial deletion group, 12) were enrolled, and 30 CNS HGBs from these patients were studied. Clinical data showed that men at first operation were significantly younger than females (*p* = 0.005). Real-time PCR demonstrated that *EGFR* (*p* = 0.017) and *TGF*α (*p* = 0.017) mRNA expression in VHL-related HGBs was significantly higher than that in the control group. Immunohistochemistry showed that the mean optical density in VHL-related HGBs was significantly higher than that in controls (EGFR, *p* = 0.007; TGFα, *p* = 0.021). Finally, the cyst volume was related to the upregulation of EGFR (*r* = 0.782, *p* < 0.01).

**Conclusion:** Overexpression of EGFR and TGFα may contribute to tumor growth in VHL-related CNS HGBs. The cyst volume was associated with EGFR overexpression. These results provide information for the management of VHL-related HGBs in the era of targeted therapeutics.

## Introduction

Central nervous system (CNS) hemangioblastoma (HGB) is a benign tumor, which often occurs in the cerebellum and spinal cord, and it is the most frequent cause of mortality in patients with von Hippel–Lindau (VHL) disease (1, 2). More than 80% of VHL patients develop a CNS HGB during their lifetime (3, 4). VHL disease is an autosomal dominant genetic disease caused by germline mutations in the *VHL* tumor suppressor gene on chromosome 3 (3p25-26). Mutations in the *VHL* gene lead to inactivation of the tumor suppressor protein pVHL, which regulates the hypoxia-inducible factor (HIF) protein. Uncontrolled HIF expression caused by pVHL inactivation increases the expression of a wide range of target genes including vascular endothelial growth factor (VEGF) and C–X–C motif chemokine receptor 4 (CXCR4) (5–7).

A growing number of studies have demonstrated that the receptor tyrosine kinase (RTK) turnover plays an important role in downstream signaling in VHL disease, especially in the regulation of the epidermal growth factor receptor (EGFR) and transforming growth factor alpha (TGFα) autocrine or juxtacrine loop (8, 9). Inactivation of pVHL leads to uncontrolled HIF expression that can lead to upregulation of TGFα. TGFα binds to the EGFR receptor and is a potent angiogenic factor, forming an autocrine or juxtacrine loop to promote tumor progression in VHL-associated renal cell carcinoma (10, 11). Overexpression of TGFα and EGFR is also detected in a variety of human cancers, including epithelial and lung cancers, and gliomas (12–14). Some studies have demonstrated that the TGFα/EGFR autocrine loop plays an important role in polycystic kidneys and pancreatic neoplasms (15, 16). However, at present, the role of EGFR and TGFα in VHL-associated hemangioblastomas has not been examined. To investigate this possible role, we analyzed the relationship between the protein and mRNA expression of EGFR and TGFα in 30 CNS HGBs from 21 patients with VHL syndrome, confirmed by genetic testing. Furthermore, the correlation between the differentially expressed proteins, histological grading, genetic mutations, and tumor burden were also analyzed.

## Materials and Methods

### Patients and Tissue Samples

This study was approved by the Ethics Committee of our hospital. All patients involved in this study provided informed consent prior to the initiation of the study. From September 2015 to February 2019, 21 patients (male, *n* = 11; female, *n* = 10) with VHL, confirmed by genetic diagnosis, underwent surgery for CNS HGBs in our hospital. Thirty CNS HGBs were surgically removed, all of which were verified by postoperative pathology. These tumor tissues were embedded in paraffin or frozen directly and stored at −80°C.

### Clinical Assessment and Image Analysis

All 21 patients underwent contrast-enhanced magnetic resonance imaging (MRI) examinations (T1-weighted imaging [T1WI], T2-weighted imaging, diffusion-weighted imaging, and contrast-enhanced T1WI; slice thickness, 1 mm) to determine the lesion location. The volume of the tumor and associated cysts was calculated by using a Picture Archiving and Communication System containing a DICOM viewer software (CARESTREAM PACS, CarestreamHealth Inc, Toronto, Canada). All enrolled patients were additionally examined with abdominal computed tomography and fundoscopy to identify related lesions in other target organs. The Karnofsky Performance Scale (KPS) score was used to evaluate neurological function. Postoperative follow-up examinations were performed every 6 months. Patients underwent follow-up contrast-enhanced MRI examinations to determine the presence of unresected tumor or recurrence.

### Germline Genotype Analysis

Genomic DNA from peripheral blood was extracted using a QIAamp DNA Blood Mini Kit (QIAGEN, Germany). Three coding exons and their flanking intronic regions were amplified by polymerase chain reaction (PCR) using primers described previously (17). Missense and splicing mutations were detected by direct sequencing. A multiplex ligation-dependent probe amplification kit (MLPA, P016-C2, MRC-Holland, Amsterdam) was used to detect the large exon deletions, which were confirmed by Real-time (RT) quantitative PCR with primers described by Ebenazer et al. (18). All patients were divided into two groups: the missense group and the partial deletion group.

### Immunohistochemistry

The paraffin-embedded tissue from 30 VHL-related CNS HGBs (medulla oblongata, *n* = 5, cerebellum, *n* = 25) and three control brains from cerebral hemorrhage patients was cut at a thickness of 4 μm Paraffin-embedded sections were deparaffinized in xylene and rehydrated through a graded series of alcohols. Heat-induced antigen retrieval was performed in 10 mM citrate buffer, pH 6.0 for 10 min in an 800-Watt microwave. After cooling, sections were incubated in 3% H_2_O_2_ for 10 min at room temperature to quench endogenous peroxidases. Next, sections were incubated in 5% bovine serum albumin (BSA) for 30 min to block non-specific binding. Sections were then incubated with a rabbit anti-human phospho EGFR (1:200, Abcam, ab40815) and a rabbit anti-human TGFα (1:200, Abcam, ab208156) at 4°C overnight. After rinsing, a rabbit-specific horseradish peroxidase (HRP) polymer kit (Beijing Zhongshan Jinqiao Biotechnology, PV-9001) was used at room temperature for 50 min and the antibody reaction was visualized with chromogen 3,3-diaminobenzidine tetrahydrochloride (DAB). Sections were counterstained with hematoxylin and mounted with DPX after dehydration.

The expression of EGFR and TGFα among these sections was evaluated by measuring integral optical density (IOD) and mean optical density (MOD) using the Image-Pro Plus 6.0 (Media Cybernetics, Rockville, MD, USA) (19). The following scoring method for the assessment of EGFR and TGFα was used: score 0 ≤ 10% stained tumor cells; score 1 ≥ 10% but <25% stained tumor cells; score 2 ≥ 25% but <50% stained tumor cells; and score 3 ≥ 50% stained tumor cells (20).

### RT PCR

Total RNA was extracted from 7 snap-frozen CNS HGBs and 3 control brains from cerebral hemorrhage patients using a RNeasy kit (Invitrogen, St. Louis, MO, USA). The RNA was reverse transcribed with a first strand cDNA synthesis kit (MBI Fermentas, Lithuania). RT PCR was used to quantitatively analyze RNA expression using the Premix Taq™ (Ex Taq™ Version 2.0). Related data acquisition and processing was completed with a Roche LightCycler® 480II PCR machine (Roche, Switzerland). The values of cycle threshold (CT) for theses samples was normalized to the corresponding GAPDH CT values. The relative expression of RNA was assessed with the ΔΔCT method.

### Statistical Analysis

Patients and tumor characteristics were analyzed using descriptive statistics. The difference between HGBs and controls for the MOD and ΔΔCT values was compared using the Mann–Whitney U test. The immunohistochemical score and the age at first operation between the partial deletion group and missense groups were also compared using the Mann–Whitney U test. The Chi-squared test was used to compare the sex distribution between groups. The relation between immunohistochemical score and cyst size was evaluated by linear regression. Significance was assumed for two-sided *p*-values < 0.05. The data were analyzed with the Statistical Package for Social Science (SPSS) 21.0 (IBM, Armonk, NY, USA).

## Results

### Patient Characteristics and Tumor Burden

A total of 21 VHL patients confirmed by genetic testing (missense group, *n* = 9; partial deletion group, *n* = 12) from different pedigrees of 12 Chinese provinces were enrolled in this study. The mean age (SD) was 33.3 ± 10.2 years (range, 21–58; median, 31). The male-to-female ratio was 1.1 (males, *n* = 11; females, *n* = 10). All patients had received 44 operations for CNS HGBs, with an average of 2.1 ± 1.4 operations (range, 1–5; median, 1). The mean age at first operation in these patients was 28.7 ± 11.8 years (range, 13–58; median, 27). Our results showed no significant difference in sex (*p* = 0.806), age at first operation (*p* = 0.24), or number of operations (*p* = 0.937) between the missense group and the partial deletion group. However, the age at first operation of men was found significantly lower than that of women (*p* = 0.005).

The preoperative MRI showed that the 21 patients harbored 116 CNS HGBs on admission, with an average of 5.5 ± 3.1 tumors/patient (range, 2–13; median, 4). Because of obvious occupying effects, 18 cystic tumors and three solid tumors were resected. Because of their proximity to the larger tumors, another nine solid tumors were also removed at the same time to prevent an increase in postoperative volume. The average volume of a resected cyst from HGBs was 16.9 ± 10.7 cm^3^ (range, 1.4–41.3; median, 18.7). The cyst volumes showed no difference in sex (*p*= 0.161) and mutation type (*p* = 0.436). Among the 21 patients, 90% had pancreatic cysts, 14% had pancreatic neuroendocrine tumors, 57% had renal cysts, 38% had renal cancer, and 29% had pheochromocytomas. Retinal HGBs occurred in 43% of patients.

Twenty patients recovered well postoperatively and were discharged ~1 week later. One patient died from aspiration after surgery. Because of the high recurrence and low growth rate, our follow-up interval was 6 months. The mean follow-up duration in these 21 patients was 2.05 ± 1.04 years (range, 0.5–4; median, 2). The KPS score at 6 months postoperatively was 95 ± 7.6 (range, 80–100; median, 100).

### Immunohistochemistry

The expression of EGFR and TGFα was analyzed in tissue from 18 cystic and 12 solid HGBs with immunohistochemistry. Twenty-five tumors were located in the cerebellum and five in the medulla oblongata, while tissue from three normal brains was used as negative control. The results of Image-Pro Plus for EGFR and TGFα showed that the MOD in VHL-related HGBs was significantly higher than that in the control (EGFR, *p* = 0.007; TGFα, *p* = 0.021).

**Figure 1:**
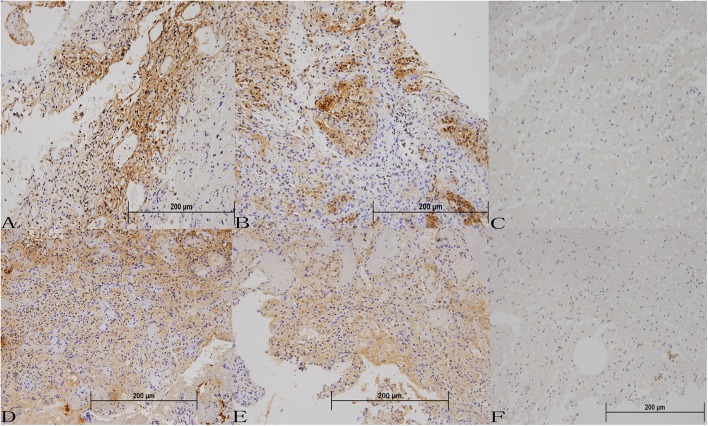
Protein expression of EGFR and TGFα in VHL-related hemangioblastoma tissues **(A–C)** Immunohistochemical staining for EGFR in VHL-related hemangioblastoma tissues (200 ×). **(D–F)** Immunohistochemical staining for TGFα in VHL-related hemangioblastoma tissues (200 ×).

The immunoreactivity for pEGFR was strong in stromal cells in all of the VHL-related CNS HGBs. Immunoreactive products were mainly located in the cytoplasm and along the cell membrane of the stromal cells. TGFα expression was also observed in the cytoplasm. However, local differences in the intensity of immunostaining are observed in most tumors. The staining intensity for TGFα appeared to be weaker than that of EGFR (Figure 1). However, tissue from two of the tumors appeared to be negative in TGFα expression compared to normal brain tissue. The mean positive cell percentage of EGFR in 30 tumor tissues was (68.6 ± 14.7)%. And the TGFα was (61.9 ± 26.8)%. The immunohistochemical score revealed that most of the HGBs had strong positive expression for EGFR and TGFα (Table 1), while there was no difference in expression between the partial deletion group and the missense group (TGFα, *p* = 0.435; EGFR, *p* = 0.539). Our analysis also showed that the expression intensity of EGFR and TGFα in relation to the solid tumor size had no statistical difference. The mean immunohistochemical positive cell percentage of EGFR had a positive correlation with the cyst volume (r = 0.782, *p* < 0.01). However, the combination of EGFR and TGFα expression levels or the TGFα immunohistochemical score showed no correlation with the cyst volume.

**Table 1:** Immunohistochemistry score for EGFR and TFGα in different mutation groups.

	**Grading**	**EGFR**	**TGFα**
Partial deletion	3	17	14
	2	2	3
	0	0	2
Missense	3	9	6
	2	2	5

### RT PCR

**Figure 2:**
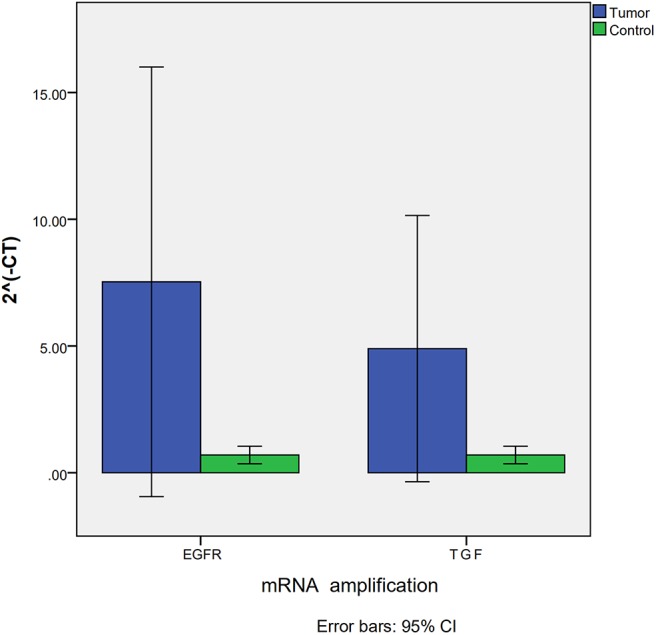
Expression of *EGFR* and *TGF*α mRNA assessed by RT-PCR showing significant upregulation for both *EGFR* and *TGF*α transcripts in VHL-related hemangioblastomas.

Due to the deficiency of fresh-frozen tissue, only tissue from seven VHL-related HGBs and three controls were used in RT PCR to detect expression of *EGFR* and *TGF*α mRNAs. The results were similar to those obtained with the immunohistochemistry analysis. *EGFR* and *TGF*α mRNAs were upregulated in all the tumors compared to the control group. ΔΔCT analysis revealed that the expression of *EGFR* (*p* = 0.017) and *TGF*α (*p* = 0.017) mRNAs in VHL-related HGBs was significantly higher than that of the control group (Figure 2).

## Discussion

EGFR is a 170 kD transmembrane glycoprotein, which belongs to the RTK family. Binding of EGFR with its ligands TGFα or EGF results in autophosphorylation of RTK followed by activation of downstream pathways, including the RAS pathway, that regulate cell proliferation and differentiation in various types of cancer. Our results revealed that overexpression of pEGFR was found in all VHL-associated HGBs. This is in line with results of previous studies demonstrating EGFR overexpression in CNS HGBs. However, none of the patients studied previously had undergone genetic testing for detecting *VHL* mutations (21, 22). Here, we analyzed genetic mutations in all 21 VHL patients who underwent a CNS HGB operation at our hospital. The results showed that overexpression of EGFR was found in patients with different types of genetic mutations in the *VHL* gene. In addition, expression intensity of EGFR showed no difference between the partial deletion group and missense group. However, the expression intensity of EGFR was stronger than that of TGFα. This is in contrast to previous findings showing that activated HIF, caused by VHL loss, upregulates TGFα expression (11). Other studies have reported that activated EGFR is associated with the pVHL-dependent polyubiquitylation and proteasomal degradation pathway (23). For this reason, activated EGFR without degradation caused by inactivation pVHL might lead to a stronger expression intensity in immunohistochemistry assays.

Immunohistochemistry analysis revealed that the patients' cyst volume was related to the upregulation of EGFR (*r* = 0.532, *p* = 0.023). This is in line with results showing that the TGFα and EGFR autocrine loops provide a platform for renal-cyst formation. The EGFR/TGFα autocrine loop constantly promotes the growth of the endothelium (24). Abnormalities in extracellular-matrix formation and vascular permeability lead to excess interstitial fluid infiltration and the initiation of cyst formation. VHL patients usually have multiple organ cystic changes such as renal and pancreatic cysts (25). In our patients, 90% had pancreatic cysts and 57% renal cysts. The main reason for operating the CNS HGBs was the mass occupying effect caused by changes in the cystic tumors, especially in the cerebellum. Because the activation of EGFR and TGFα autocrine loop occurs in changing cysts of multiple target organs, this pathway might be exploited as a potential target for anti-tumor therapy for VHL patients.

Upregulation of VEGF is a leading factor in tumor angiogenesis, especially in HGBs. Because of genetic mutations, most VHL patients develop multiple CNS HGBs and undergo multiple surgeries. Hence, targeted therapy as a supplement to surgical treatment is deemed necessary. The main target therapy for HGBs is anti-angiogenic agents. However, clinical trials for retinal HGBs demonstrated that an anti-VEGF agent resulted in edema reduction and improved visual acuity, without changing the tumor size. Once the treatment was terminated, patients had repeated symptoms due to aggravation of the edema (26, 27). Hence, other target therapies of CNS HGBs are necessary. Rogers et al. used the Erlotinib therapy (ATP competitive inhibitor of tumor-cell EGFR tyrosine kinase) for VHL-related CNS HGBs in one patient, achieving good results (28). An inhibitor of EGFR tyrosine kinase was also effective as a treatment for polycystic kidney disease by reducing cystic lesions (29, 30). Therefore, the EGFR family of RTKs might be exploited as a potential target for integrated therapy in VHL patients.

## Conclusions

Overexpression EGFR and TGFα may contribute to tumor growth in VHL-related hemangioblastomas. Overexpression of EGFR was present in a variety of VHL mutations, and the volume of the cyst was related to the upregulation of EGFR (*r* = 0.532; *p* = 0.023). The current results provide information for the management of VHL-related HGBs in the era of targeted therapeutics.

## Data Availability Statement

The datasets generated for this study are available on request to the corresponding author.

## Ethics Statement

The studies involving human participants were reviewed and approved by The Institutional Review Board of Peking University First Hospital (Beijing, China). The patients/participants provided their written informed consent to participate in this study. Written informed consent was obtained from the individual(s) for the publication of any potentially identifiable images or data included in this article.

## Author Contributions

ZL were responsible for the concept and design of the study. LL and ZY dealt with the clinical data. HD and RL performed the statistical work. CL, JZ, and KG provides the figures and tables. All authors revised the manuscript.

## Conflict of Interest

The authors declare that the research was conducted in the absence of any commercial or financial relationships that could be construed as a potential conflict of interest.

## Abbreviations

EGFR: epidermal growth factor receptor
HGB: hemangioblastoma
HIF: hypoxia-inducible factor
KPS: Karnofsky Performance Scale
MOD: mean optical density
RTK: receptor tyrosine kinase
TGFα: transforming growth factor alpha
VHL: von Hippel–Lindau.
